# Department of Medicine in the University of Michigan

**Published:** 1849

**Authors:** 


### ARTICLE III.

### DEPARTMENT OF MEDICINE IN THE UNIVERSITY OF MICHIGAN.

We observe by the catalogue of the "College of the Arts and Sciences of the University of Michigan," that it is contemplated to commence a course of instruction in the department of Medicine, next autumn.

"The Board of Regents have adopted the plan of requiring but one course of lectures, which, in consequence, will be extended through the academic year, and subject to the same vacations as in the department of Science and Arts."

There are three vacations annually, viz.:—"From the third week in July, eight weeks; from Wednesday, next preceding 25th December, two weeks; from the third Wednesday in April, three weeks."

Students are to undergo an examination for admission, and will be required to come up to the standard of preliminary education of the National Medical Convention.

The expenses will be the same as those in the department of Science and Arts. An announcement will be published in the course of the year. E.

